# Characterization of spermidine hydroxycinnamoyl transferases from eggplant (*Solanum melongena* L.) and its wild relative *Solanum richardii* Dunal

**DOI:** 10.1038/hortres.2016.62

**Published:** 2016-12-07

**Authors:** Hui Peng, Tianbao Yang, Bruce D Whitaker, Frances Trouth, Lingfei Shangguan, Wen Dong, Wayne M Jurick

**Affiliations:** 1 Food Quality Laboratory, Beltsville Agricultural Research Center, Agricultural Research Service of U.S. Department of Agriculture, Beltsville, MD 20705, USA; 2 Horticulture & Landscape College, Hunan Agricultural University, Changsha 410128, Hunan, China; 3 College of Horticulture, Nanjing Agricultural University, Nanjing 210095, Jiangsu, China; 4 Department of Plant Science, University of Tennessee, Knoxville, TN 37996, USA

## Abstract

Eggplant produces a variety of hydroxycinnamic acid amides (HCAAs) that have an important role in plant development and adaptation to environmental changes. In this study, we identified and characterized a spermidine hydroxycinnamoyl transferase (SHT) from eggplant (*Solanum melongena*) and its wild relative *S. richardii*, designated as *SmSHT* and *SrSHT*, respectively. *SmSHT* was abundant in flowers and fruits, whereas the level of *SrSHT* was remarkably low in all tissues. Heat-shock/drought treatment stimulated the expression of *SmSHT* in both leaves and fruits, indicating its involvement in plant stress response. Both SHT polypeptides had extremely high identity with just five amino-acid substitutions. Recombinant SmSHT catalyzed the synthesis of mono-, bi- and tri- acylated polyamines. Using caffeoyl-CoA as the acyl donor, SmSHT preferred spermidine as the acyl acceptor. When spermidine was the acyl acceptor, the donor preference order for SmSHT was caffeoyl-CoA>feruloyl-CoA>*ρ*-coumaroyl-CoA. SrSHT exhibited the same substrate specificity as SmSHT, yet exhibited significantly higher catalytic activity than SmSHT. For example, under caffeoyl-CoA and spermidine, *K*_cat_ of SrSHT was 37.3% higher than SmSHT. Molecular modeling suggests that five amino-acid substitutions in SrSHT result in four alterations in their predicted 3D structures. In particular, the conserved Lys402 adjacent to the DFGWG motif, and Cys200 in the crossover loop in SmSHT were replaced by Glu and Ser in SrSHT. These substitutions may contribute to the enhanced activity in SrSHT. Our study provides a platform to generate HCAA rich fruits for eggplant and other solanaceous crops.

## Introduction

**Figure 1:**
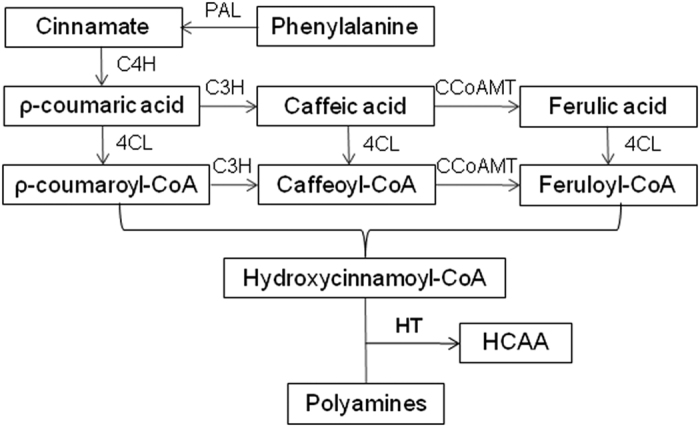
HCAA biosynthesis pathway in plants. PAL, phenylalanine ammonia lyase; C4H, cinnamate 4-hydroxylase; C3H, cinnamate 3-hydrolase; 4CL, 4-coumaroyl-coenzyme A ligase; CCoAOMT, caffeoyl-CoA o-methyltransferase; HCSpd, hydroxycinnamoyl spermidine; HT, polyamine hydroxycinnamoyl transferase.

Hydroxycinnamic acid amides (HCAAs)^1^ have a critical role in plant growth and developmental processes, including cell division, cytomorphogenesis, flowering, cell wall cross-linking, tuberization and stress responses.^2–6^ They are also beneficial to human health since these compounds have anticarcinogenic, antihypertensive, antimicrobial and other potentially therapeutic activities.^7–11^ HCAAs are derived from the phenylpropanoid pathway (Figure 1). The first step in the biosynthetic pathway is that l-phenylalanine is converted to *trans*-cinnamate by phenylalanine ammonia lyase (PAL). *Trans*-cinnamate is then hydroxylated to form ρ-coumaric acid by 4-hydroxylase (C4H) and converted to other hydroxycinnamates such as caffeic acid, ferulic acid and sinapic acid.^12,13^ These hydroxycinnamates are formed into the corresponding CoA esters by 4-cinnamate: CoA ligase (4CL) and serve as substrates for entry into various branch pathways such as HCAA, flavonoid, and lignin biosyntheses.^14^ For HCAAs biosynthesis, the ultimate and committed step is the condensation of hydroxycinnamoyl-CoA thioesters with polyamines such as spermidine (Spd), spermine (Spm) and putrescine (Put) catalyzed by hydroxycinnamoyl transferases.^15^

Spermidine hydroxycinnamoyl transferase (SHT) belongs to the acyl-CoA-dependent BAHD super family. The members of this family are responsible for biosynthesis or modification of diverse metabolites such as alkaloids, terpenoids and phenolics.^16^ They possess a conserved catalytic HXXXD domain and a functionally unknown DFGWG motif.^16^ Several SHTs have been isolated and characterized in plants.^5^ An *Arabidopsis thaliana* SHT (AtSHT) was shown to catalyze the formation of mono-, di- and tri- acylated-Spd.^17^ Some other SHTs in *Arabidopsis* regulate the accumulation of disinapoyl-Spd and dicoumaroyl-Spd in seeds.^4^ A tobacco SHT (NaDH29) catalyzes the synthesis of hydroxycinnamoyl spermidine (HCSpd).^18^ Although, all these enzymes prefer Spd as acyl donor substrate, they display distinct catalytic specificities and activities. Crystal structures of SHT related BAHD proteins^19,20^ revealed there are several critical sites for substrate binding and catalytic activity. However, the structure and functional relationship of SHTs has not been studied.

Eggplant (*Solanum melongena* L.), as a commercially important vegetable, contains abundant and diverse phenolic products that are beneficial to human health.^21^ By means of high-antioxidant activity, eggplant has been ranked among the top 10 for scavenging superoxide ions among120 vegetables.^22^ This is attributed to high phenolic content including HCAAs in the flesh.^23–26^ *S. richardii*, a wild relative of eggplant from Africa, is rich in HCAAs although their composition and the relative abundance of HCAAs are substantially different from cultivated eggplant.^23,25^ To date, HCAA biosynthesis and regulation remains largely uncharacterized in *Solanum* species.^27,28^ Here we report the isolation and characterization of a novel SHT from eggplant and its ortholog from *S. richardii*. The structural features, substrate specificity and catalytic activities of both SHTs were analyzed.

## Materials and methods

### Plant growth and heat shock/drought treatment

Eggplant (*S. melongena* L. cv Black Beauty) and its African wild relative (*S. richardii* Dunal, USDA Agricultural Research Service germplasm accession PI500922) were grown in a greenhouse at 28 °C with 16 h/8 h (light/dark) photoperiod.

Fully opened young eaves, open flowers, young fruits (10 days post anthesis) and mature fruits (20 days post anthesis) were collected for gene expression analysis. For heat shock/drought treatment, fully opened leaves and mature fruits were harvested and laid on table at room temperature for 1 h. Then half samples were kept at room temperature, and another half were transferred to an incubator at 39 °C. After treatment, leaves were collected at 0, 0.5, 2 and 4 h, and mature fruits were collected at 0, 4 and 24 h. Harvested samples were immediately frozen in liquid nitrogen and stored at −80 °C.

### Cloning of *4CL* and *SHT* genes

Total RNA was isolated from frozen fruit tissues using the RNeasy Plant Mini Kit (Qiagen, Germantown, MD, USA) according to the manufacturer’s instructions. The full-length open reading frame (ORF) of *Sm4CL1* was amplified by the high fidelity *Pfx* DNA Polymerase (Invitrogen, Frederick, MD, USA) using the gene-specific primer pair (5′-CACCATGCCGATGGATACCGAAACAAAG-3′/5′-ATTTGAAATACCAGCAGCCAGTCT-3′). Using a gene-specific primer pair (5′-ATGAAAGTTATCTTAAAGAATCATTG-3′/5′-ACATTCAATATCTTCATAGAAAAATTT-3′), *SmSHT* and *SrSHT* coding regions (CDS) were amplified from RNA in mature eggplant and *S. richardii* fruits, respectively. PCR products were ligated with TA cloning plasmid pCR4-TOPO (Invitrogen, Frederick, MD, USA) and verified by DNA sequencing (Iowa State University, Ames, Iowa, USA). Full-length *SmSHT* and *SrSHT* ORFs were deposited in GenBank with accession numbers KP165410 and KP165411, respectively.

### RT-qPCR analysis

Total RNA isolated from frozen tissues were further treated with TURBO DNase (Invitrogen, Frederick, MD, USA) to remove genomic DNA according to the manufacture’s protocol and then quantified by a NanoDrop 1000 spectrophotometer (Thermo Scientific, Waltham, MA, USA). The first strand of cDNA was obtained from RNA samples of *S. melongena* and *S. richardii* using iScript Transcription Supermix (Bio-RAD, Hercules, CA, USA). The primer pairs, 5′-CGCCAACGTGGACTGGCACTGTTT-3′/5′-CGCAAACGGCCAGCCAATGGATAGA-3′ and 5′-CCGCTCCTAGCAAAGATGCC-3′/5′-ACCCTCCACAATGCCAAACC-3′, were used for the expression analysis of *SrSHT* and *SmSHT*, and a housekeeping gene *GAPDH* (Genbank accession number JX448343),^29^ respectively. The following thermal cycle was used in real time PCR: 95 °C for 2 min, followed by 40 cycles of 95 °C for 5 s, 60 °C for 15 s. Relative quantification of specific mRNA levels was analyzed using the cycle threshold (Ct) 2^−ΔΔCt^ method. Relative expression levels are normalized using the expression of GAPDH and shown in fold changes (lowest value=1). Student’s *t*-test (*P*<0.05) was used to determine the significant difference of relative expression of individual genes among different tissues (Microsoft Excel 2010). All experiments were repeated three times.

### Bioinformatics analysis and *in silico* structural modeling

Multiple sequence alignments were performed using ClustalW in Geneious Pro (4.8.5) (http://www.geneious.com/). Phylogenetic analysis was performed with the Neighbor-Joining (NJ) method using Geneious Pro (4.8.5) with 1000 bootstrap replications. The homology model of SHT was constructed using the Swiss-model (http://swissmodel.expasy.org) based on the crystal structure of a coffee hydroxycinnamoyl transferase CcHCT (pdb code: 4G0B).^19^ The model and the template were analyzed using the Swiss PDB viewer (http://spdbv.vital-it.ch/).^30^

### Expression and purification of recombinant 4CL and SHT proteins

Sm4CL1 CDS was directly cloned into pET102/D-TOPO expression vector (Invitrogen, Frederick, MD, USA) in frame with a C-terminal His-tag. Both SHT cDNAs were inserted into *Nco*I and *Eco*RI restriction sites of pET-32a(+) expression vector (Millipore, Gibbstown, NJ, USA) in frame with N-terminal His-tag. Expression of target proteins was induced by isopropyl β-D-1-thiogalactopyranoside (IPTG). The cells were then collected and resuspended in Tris-lysis buffer. The supernatant was applied to Ni-NTA Superflow resins (Qiagen, Germantown, MD, USA) and the recombinant proteins were purified following the manufacturer’s instructions.

### Enzyme activity assays

4CL activity was assayed as described.^31^ Cinnamate substrates included caffeic, ferulic, coumaric and sinapic acids (Sigma-Aldrich, St Louis, MO, USA). The reaction was conducted at 30 °C for 1 h after adding recombinant Sm4CL1 protein.

The acylation reaction was performed according to Hedberg *et al.*^15^ with minor modification. The standard acylation assay contained 120 μm acyl donor (hydroxycinnamoyl-CoA) and 2.5 mm acyl acceptor (polyamine) in 0.1 m Tris-HCl buffer (pH=9.0). After incubating at 30 °C for 15 min, the reaction was stopped by adding one volume of 0.4% phosphoric acid. Then, the microplates were read at 342, 354 and 358 nm for monitoring ρ-coumaroyl-CoA, feruloyl-CoA and caffeoyl-CoA content, respectively, with EON microplate reader (BioTek, Winooski, VT, USA). Wells containing all other components except for polyamine served as blank controls. Standard curves were used to determine the conversion factor between the absorbance and the hydroxycinnamoyl-CoA contents. The kinetic constants for SHTs were determined using 0 to 240 μm different hydroxycinnamoyl-CoAs at 2.5 mm of acyl acceptor substrate (spermine) in the reaction mixture described above. All the reactions were run in triplicate, and each experiment was repeated at least three times.

### HPLC-DAD analysis of hydroxycinnamoyl-CoAs and HCAAs

For HPLC analysis of Sm4CL1 reaction, samples were fractionated on 1 g, 33 μm Strata X polymeric reverse phase, solid phase extraction (SPE) tubes (Phenomenex, Torrance, CA, USA) with a step gradient of pH 8 aqueous Ammonium Hydroxide, 80% aqueous MeOH and 100% MeOH. Analysis of aliquots by spectral pattern using a Shimadzu UV160U spectrophotometer showed the 80% MeOH SPE fraction was enriched for the hydroxycinnamate CoA acids. This fraction was evaporated under nitrogen to yield two 1.5 mL samples in 0.2% aqueous H_3_PO_4_, which was purified using a HP 1200 Series HPLC system (Agilent Technologies, Wilmington, DE, USA). Fifty to 100 μL portions were injected onto a Phenomenex Luna C18(2) analytical column (250 mm, 5 μm particle size, 4.6 mm intradermally (i.d.)) (Phenomenex). To achieve separation of the major constituents, caffeoyl-CoA, ferruloyl-CoA and ρ-coumaroyl-CoA, a binary mobile phase gradient of acetonitrile in aqueous 0.2% H_3_PO_4_ was used. Hydroxycinnamoyl-CoA fractions were collected (caffeoyl-CoA, 17.7 min; ferruloyl-CoA, 20.1 min; ρ-coumaroyl-CoA, 19.1 min), neutralized with NH_4_OH, placed on a nitrogen evaporator to remove all traces of acetonitrile, and salts were removed by solid phase extraction on a 500 mg Strata X, polymeric reverse phase extraction tube to yield four 1 mL samples in water at 1 nmol μL^−1^ each.

For HPLC analysis of SHT samples, each reaction was fractionated on a 60 mg, 33 μm Strata X polymeric reverse phase, solid phase extraction tube with a step gradient of pH 8 aqueous Ammonium Hydroxide, 25% aqueous MeOH, 0.1% HCl in 100% MeOH and 100% MeOH. Analysis of aliquots by spectral pattern using a Shimadzu UV160U spectrophotometer showed the 0.1% HCl in 100% MeOH SPE fraction was enriched for hydroxycinnamoyl-amides. This fraction was processed under nitrogen evaporation to yield a 1.0 mL sample in 5% aqueous MeOH, which was purified using a HP 1100 Series HPLC system (Agilent Technologies). Fifty to 100 μL portions were injected onto a Kinetex C18 100A analytical column (250 mm, 5 μm particle size, 4.6 mm i.d.; Phenomenex). To achieve separation of the major constituents, a binary mobile phase gradient of MeOH in aqueous 1% H_3_PO_4_ was used. Hydroxycinnamoyl-amide fractions were collected, neutralized with NH_4_OH, placed on a nitrogen evaporator to remove all traces of MeOH, and salts were removed by solid phase extraction on a 60 mg Strata X, polymeric reverse phase extraction tube, as previously described in this section to yield one 5% aqueous MeOH sample which was analyzed by mass spectroscopy

### Enzyme kinetic analysis

Kinetic data were fitted to the Michaelis–Menten equation.

$$v=\frac{V_{\max}\left[S\right]}{K_{m}+\left[S\right]}$$

Where [S] is the concentration of the varied substrate, *V*_max_ represents the maximal rate, and *K*_m_ is the substrate concentration at which the reaction rate is half of *V*_max_. Turnover number *K*_cat_ equals *V*_max_/[E], in which [E] is the enzyme molar concentration. *K*_cat_/*K*_m_ is used to describe catalytic efficiency of enzyme. All data were fit with KaleidoGraph version 4.5 from Synergy Software (www.synergy.com). The kinetic parameters were derived from at least three determinations. Standard variance analyses and significance analysis of difference (*t*-test, *P=0.05*) were performed in Microsoft Excel 2010.

## Results

### Synthesis of hydroxycinnamoyl-CoA’s using Sm4CL

**Figure 2:**
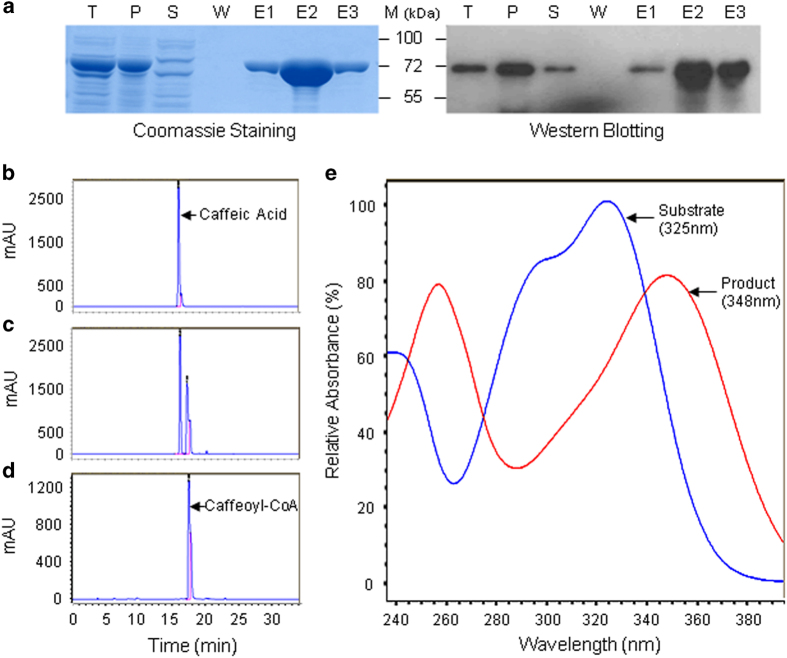
Synthesis of caffeoyl-CoA via *Solanum melongena* 4-coumarate:CoA ligase 1 (Sm4CL1). His-tagged recombinant Sm4CL1 protein was expressed in *E. coli* and purified from cell lysates by nickel affinity chromatography. (**a**) Evaluation of protein purity was performed via SDS-PAGE stained with Coomassie Brilliant Blue (left), and Western blotting using anti-His-tag antibody (right): T, total cell lysate; P, cell pellet; S, supernatant; W, last column wash; E1, E2 and E3, respectively, first, second and third column elutes. (**b**), (**c**) and (**d**) in order: C18-HPLC-DAD chromatograms of a caffeic acid standard, the substrate plus reaction product of Sm4CL1, and purified caffeoyl-CoA from the Sm4CL1 reaction (mAU, milli-absorbance units). (**e**) UV absorbance spectra of caffeic acid (substrate) and caffeoyl-CoA (product).

Owing to the lack of a commercial source of hydroxycinnamoyl-CoAs, we synthesized them by converting hydroxycinnamates to CoA esters using a recombinant eggplant 4CL enzyme *in vitro*. The eggplant genome database was searched using the BLAST program against tomato Sl4CL1 (accession no.NC_015440), a well-characterized CL1 enzyme. A total of four 4CLs were found and designated as *Sm4CL1*, *Sm4CL2*, *Sm4CL3* and *Sm4CL4*. *Sm4CL1* was selected because it had the highest homology with *Sl4CL1* (Supplementary Figure 1).^32^ DNA sequencing revealed that *Sm4CL1* encoded a putative protein of 545 aa with 96.2 and 69.1% identity to Sl4CL1 and Arabidopsis 4CL2. Like other 4CLs, Sm4CL1 contained a typical AMP-binding domain (BoxI, SSGTTGLPKGV) and a catalysis-related motif (BoxII, GEICIRG) as well as other conserved residues associated with substrate binding and catalytic activity (Supplementary Figure 2).^13,33–35^ Further, the recombinant Sm4CL1 was successfully expressed in *Escherichia coli*, which was verified by SDS-PAGE and western blot analyses (Figure 2a). Purified recombinant Sm4CL1 protein was used to catalyze the ligation of hydroxycinnamate and CoA. HPLC analysis showed that caffeic acid (substrate) had 16.2 min of retention time, and the reaction sample contained a 17.4 min peak in addition to 16.2 min peak (Figures 2b–d). Collections of these two peaks were further analyzed by HPLC-diode array detector (DAD). The peak at 16.2 min overlapped with caffeic acid absorption of 325 nm, and the peak at 17.4 min had absorbance of 348 nm (Figure 2e), which confirmed that the caffeic acid has been converted to caffeoyl-CoA. Similarly, ferulic acid and coumaric acid were also efficiently converted to corresponding CoA esters by Sm4CL1, whereas sinapic acid was not a valid substrate under our experimental conditions (data not shown). These results indicated that Sm4CL1 encodes a functional 4-coumarate: CoA ligase. Thus, three acyl donor substrates (caffeoyl-CoA, feruloyl-CoA and *ρ*-coumaroyl-CoA) were synthesized using Sm4CL1.

### Structural features of SmSHT and SrSHT

**Figure 3:**
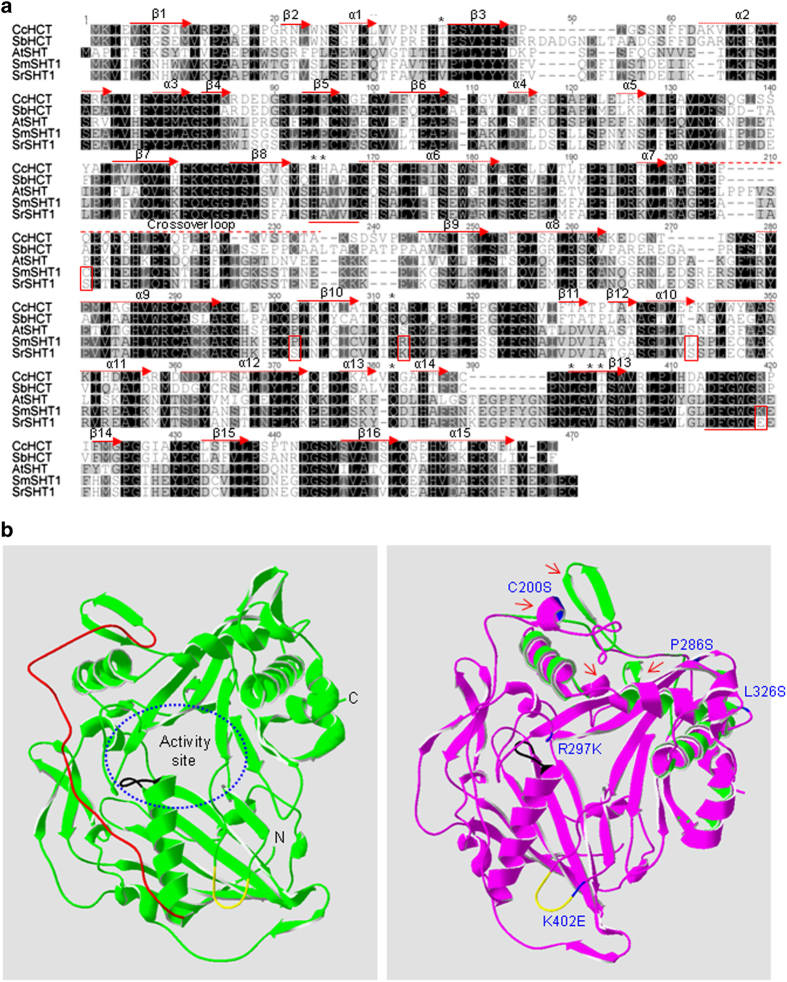
Structural features of SmSHT and SrSHT. (**a**) Amino-acid sequence alignment of SmSHT, SrSHT and three other hydroxycinnamoyl transferases. Identical and similar amino acids were shaded in black and gray, respectively. The conserved domains (HXXXD and DFGWG) in BAHD family proteins were underlined with red solid lines. A crossover loop linking N- and C-terminal domains was labeled by a red dashed line on the top. The catalytically important residues of CcHCT and SbHCT were marked with black asterisks. The α helices in CcHCT were depicted with red solid arrows and the β strands with dotted arrows. Accession numbers are as follows: AtSHT, At2g19070; CcHCT, EF137954; SbHCT, Sb04g025760; SmSHT, KP165410; SrSHT, KP165411; (**b**) Modeled 3D structures of SmSHT and SrSHT. Molecular model of SmSHT (green) and structure comparison image of SmSHT and SrSHT were shown on the left and right, respectively. Crossover loop is indicated in red. Conserved motifs HXXXD and DFGWG were labeled in black and yellow, respectively. Five substitutions between SmSHT and SrSHT were labeled in blue. All putative structure modifications were marked by red arrows.

Using the amino-acid sequence of *Arabidopsis* AtSHT against the eggplant genome database via BLAST,^36^ we found one putative ortholog with 51.1% similarity to AtSHT (Supplementary Figure 3). We cloned this gene from *S. melongena* and its ortholog from *S. richardii*, and designated them as SmSHT and SrSHT, respectively. Both putative proteins contain 453 amino acids and showed extremely high identity (98.9%) to each other. Sequence alignment revealed that only five substitutions (C200S, P286S, R297K, L326S and K402E) occurred between SmSHT and SrSHT (Figure 3a). BLAST searches in the NCBI Genbank revealed that both SHTs showed the highest similarity to the potato SHT (90.0%) and tomato SHT (88.0%). Like other BAHD family proteins, both SHTs contained typical HXXXD and DFGWG motifs (Figure 3a).^37^ Notably, most plant hydroxycinnamoyl transferases have a positively charged Lys or Arg in the position equivalent to K402 in SmSHT (Supplementary Figure 4). However, the residue adjacent to the conserved DFGWG motif was substituted by a negatively charged Glu in SrSHT (Figure 3a). C200S contained another interesting substitution because it was located in the crossover loop which links domain I and II (Figure 3a).

To determine whether amino-acid substitutions cause possible structural change, the homology models of both SHTs were constructed based on the crystal structure of CcHCT, a shikimate hydroxycinnamoyl transferases from coffee.^19^ It exhibited the highest identity (37.0%) to eggplant SHTs among the six structurally known plant BAHD proteins (Supplementary Figure 4). Similar to other BAHD super family members, the tertiary structures of SmSHT and SrSHT were composed of two nearly equal-sized domains comprising a large mixed β-sheet flanked by α-helices (Figures 3a and b). The N- and C- terminal domains were connected by a large crossover loop between Gly-177 and His-205. The catalysis-related motif HXXXD was observed to be a part of the solvent channel, although another conserved motif (DFGWG) was found to be located away from the catalytic site (Figure 3b). These results suggest that the spatial structures of BAHD family proteins are highly conserved, though they show relatively low similarity to each other at the amino-acid level. The major structural difference observed between SmSHT and SrSHT were found to be located in the C-terminal domain. Two pairs of antiparallel β sheets (Leu206–Leu214 and Ala369—Tyr378) were formed in the predicted spatial structure of SmSHT. In contrast, two short α helices (Ile198–Thr202 and Gln365–His368) were formed in SrSHT in the areas adjacent to the residues conforming β sheet pairs in SmSHT (Figure 3b).

### *SHTs* transcripts are highly expressed in cultivated eggplant fruits

**Figure 4:**
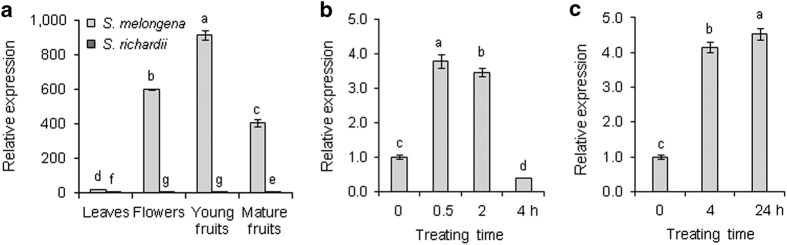
Expression analysis of *SHTs* by RT-qPCR. (**a**) Expression patterns of *SmSHT* and *SrSHT* in various tissues. (**b**) Expression of *SmSHT* in leaves under heat/drought stress. (**c**) Expression of *SmSHT* in fruits under heat/drought stress. qRT-PCR was performed in triplicates with *GAPDH* as a constitutive control. The data represent the mean value (±s.d.) of three independent biological replicates. Different letters indicate significant differences among mean values (*P*<0.05; *t*-test).

Expression of *SHTs* was investigated in wild and cultivated eggplant plants using RT-qPCR. As shown in Figure 4a, *SmSHT* transcripts in cultivated eggplant highly accumulated in flowers, and both young and mature fruits, but had low expression in leaves. The expression level of *SmSHT* in young fruits was 45 times more than that in leaves (Figure 4a). However, the expression of *SrSHT* was remarkably low in all the examined tissues in *S. richardii*. For instance, SmSHT expression level was about 80 times to that of *SrSHT* in mature fruits.

### Heat shock/drought stress stimulates *SmSHT* in leaves and fruits

Previous studies implicate that HCAA was involved in stress responses.^3,5^ To determine whether eggplant SHT has a role in stress response, we investigated its expression pattern in leaves and mature fruits under high temperature (39 °C) and drought. The expression of *SmSHT* in the incised leaves increased four times by heat shock and dehydration in 30 min, kept at relatively high level after 2 h, and then dropped to a level even lower than time 0 after 4 h (Figure 4b). For treated fruits, the stress treatment enhanced the expression of *SmSHT* more than fourfolds after 4 h, and kept the high level until time point at 24 h (Figure 4c). These results indicate that *SmSHT* is responsive to heat shock and drought stress.

### Both SHTs are functional hydroxycinnamoyl transferases

**Figure 5:**
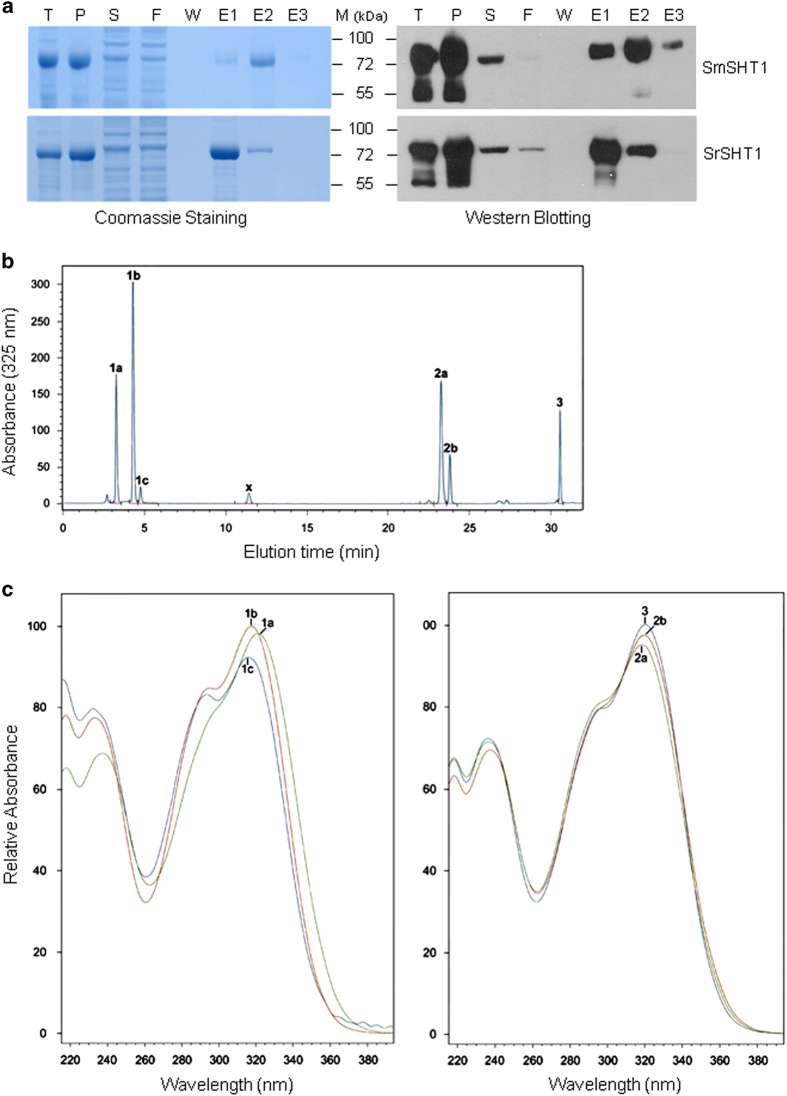
Synthesis of feruloylspermidine conjugates via recombinant *Solanum melongena* and *S*. *richardii* spermidine:hydroxycinnamoyl-CoA *N*-acyltansferase 1 orthologs (SmSHT and SrSHT). His-tagged recombinant SmSHT and SrSHT proteins were expressed in *E*. *coli* and purified from cell lysates by nickel affinity chromatography. (**a**) Evaluation of protein purity was performed via SDS-PAGE stained with Coomassie Brilliant Blue (left), and western blotting using anti-His-tag antibody (right): T, total cell lysate; P, cell pellet; S, supernatant; F, Flow through solution; W, last column wash; E1, E2 and E3, respectively, first, second and third column elutes. (**b**) C18-HPLC-DAD chromatogram of reaction products of SmSHT with spermidine plus feruloyl-CoA as substrates (mAU, milli-absorption units). (**c**) UV absorbance spectra of mono- (peaks 1a–1c), bis- (peaks 2a and 2b), and tris- (peak 3) *N*-feruloylspermidine conjugates.

To examine the enzymatic activity of SHTs, the recombinant proteins with His-tag were heterologously expressed in *E. coli*. SDS-PAGE showed overexpression of a band with a size of 73 kDa in the soluble fraction of SmSHT or SrSHT isolates. The size matched the predicted SHT size (51 kDa) of SmSHT or SrSHT plus His-tag. Western blot analysis with anti-His antibody further confirmed the major bands were the recombinant SHT proteins containing the His-tag (Figure 5a).

Activities of recombinant SHT proteins were tested with the donor substrates (caffeoyl-, feruloyl- and *ρ*-coumaroyl-CoA) purified by HPLC, as well as the potential acceptor substrates (Spd, Spm and Put). Spectrophotometric analysis showed that all of donor substrates were utilized by both enzymes with any of three polyamines (Supplementary Figures 5 and 6). Figure 5b shows an example of the analysis of feruloyl-CoA and Spd reaction catalyzed by SmSHT. There were seven peaks in the retention time chart. Peaks 1a, 1b, 1c, 2a, 2b and 3 were identical to standards of *N*^5^-feruloylspermidine, *N*^1^-feruloylspermidine, *N*^10^-feruloylspermidine, *N*^1^,*N*^5^-bi(feruloyl)spermidine, *N*^5^,*N*^10^-bi(feruloyl)spermidine and *N*^1^,*N*^5^,*N*^10^-tri(feruloyl)spermidine, respectively. In UV absorption spectra, these compounds corresponding to peaks 1a, 1b, 1c, 2a, 2b and 3 had the characteristic absorption at 322, 318, 317, 318, 320 and 322 nm, respectively (Figure 5c). Similarly, SmSHT conjugated Spm and Put with multiple acyl groups (Supplementary Figure 5). In addition, *ρ*-coumaroyl-CoA and caffeoyl-CoA also were converted to various acylated polyamines by SmSHT (data not shown). SrSHT was found to be able to function in the similar manner thus indicating that both SmSHT and SrSHT were hydroxycinnamoyl transferases (Supplementary Figures 5 and 6).

### Substrate specificity of the recombinant SHTs

Before conducting the kinetics assay, we optimized the SHT catalytic conditions including temperature (25, 30 and 37 °C), reaction time (5, 8, 11, 15 and 20 min) as well as DTT, EDTA and Mg^2+^. The initial velocity was measured with different concentrations of the substrates at several reaction time points. Both SmSHT and SrSHT exhibited the highest activity at 30 °C, and their initial velocity displayed a straight line during 5–15 min. DTT (1 mm) obviously reduced the enzyme activity, and EDTA (10 mm) and Mg^2+^ (2 mm) had no detectable effect on the enzyme activity. Therefore, we selected the optimized condition as 30 °C for 15 min with 10 mm EDTA.

**Figure 6:**
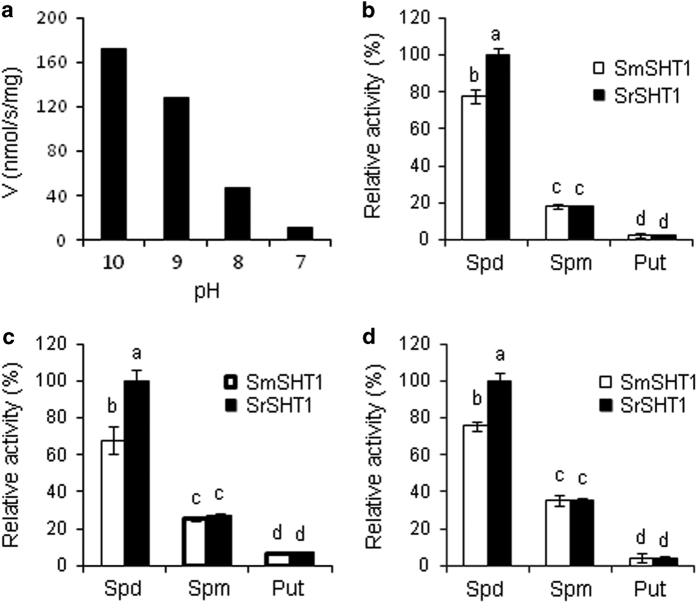
Catalytic specificity of SHTs. (**a**) pH-dependent activities of SmSHT. The activity assay was performed at 120 μm caffeoyl-CoA and 2.5 mm spermidine in 100 mm Tris buffer. (**b**–**d**) Acyl donor substrate specificity of SmSHT and SrSHT with caffeoyl-CoA, feruloyl-CoA, and ρ-coumaroyl-CoA, respectively. The activities were measured at 120 μm hydroxycinnamoyl-CoA and 2.5 mm polyamine (Put, putrescine; Spd, spermidine; Spm, spermine). All activity values were compared against the greatest value expressed as a percentage. Mean relative activity values and s.e. were calculated from duplicates. For each chart, different letters indicate significant differences among mean values (*P*<0.05; *t*-test).

To define the optimal pH for SHTs, activities of recombinant proteins were examined in the pH range of 7.0 to 10.0 with Spd and feruroyl-CoA substrates. SmSHT has no detectable activity at pH 7.0. However, its activity increased as pH rose. The activity rose over 40-fold at pH 8.0, further doubled at pH 9.0, and then slightly increased at pH 10.0 (Figure 6a). SrSHT behaved similarly during pH activity optimization (data not shown). We selected pH 9.0 for the kinetic analysis based on the reasons below. First, all the characterized SHT homologs were analyzed at pH 8.0–9.0.^4^ We could have meaningful comparison between our enzymes and others. Second, pH 9.0 is closer to the physiological pH range in plants. All examined polyamines (Spd, Spm and Put) were acylated by both enzymes, but the favored substrate for both SHT was Spd, followed by Spm and Put (Figures 6b–d). For example, when using feruloyl-CoA as a donor substrate, SrSHT only displayed 23 and 3% of activities on Spm and Put as compared with Spd (Figure 6c). At the fixed concentration of Spd, caffeoyl-CoA was the most efficient substrate for both SHTs among the three donor substrates. The specific activity of SrSHT on feruloyl-CoA and *ρ*-coumaroyl-CoA were only 74% and 50% of that on caffeoyl-CoA, respectively. However, under the fixed concentration of Spm and Put, all three acyl esters were utilized by both enzymes (Figures 6b–d). Notably, the activity of SrSHT was significantly higher than that of SmSHT, especially under the fixed concentrations of Spd. For example, the activity of SmSHT was 68% of SrSHT for conjugating feruloyl-CoA and Spd. These results indicate that both SHTs have the same substrate specificity but different catalytic capabilities.

### SrSHT displays higher activity than SmSHT

To further investigate the catalytic properties of both SHTs, the steady-state kinetics were performed at the saturated concentration (2.5 mm) of Spd, while hydroxycinnamoyl-CoA concentrations varied from 0 to 240 μm. We found that kinetics of both SHTs closely fit Michaelis–Menten curves, which was confirmed by the linearity of the Lineweaver–Burk plots (Supplementary Figure 7). The specific activity of SmSHT and SrSHT towards caffeoyl-CoA was 50.7 and 65.3 nmol s^−1^ mg^−1^, respectively, at 120 μm spermidine. The *K*_m_ values of SmSHT and SrSHT were not significantly different, indicating that both enzymes have similar affinity for this substrate. However, the turnover numbers (*K*_cat_) of SmSHT and SrSHT for caffeoyl-CoA were 441.8 and 606.8 min^−1^, respectively. Catalytic efficiency (*K*_cat_/*K*_m_) of SrSHT for caffeoyl-Spd was hence 18.4% higher than that of SmSHT (Table 1). In comparison, feruloyl-CoA displayed higher affinity to SmSHT than SrSTH1, with the *K*_m_ values at 27.3 μm for SmSHT, and at 51.3 μm for SrSHT. For feruloyl-CoA, the *K*_cat_ of SrSHT was significantly higher than that of SmSHT, although the calculated catalytic efficiency (*K*_cat_/*K*_m_) of both enzymes did not show obvious differences (Table 1). *ρ*-coumaroyl-CoA had similar affinity for both enzymes because there was no significant difference between their *K*_m_ values. At the same time, the turnover number of SrSHT was higher than that of SmSHT, suggesting that the calculated catalytic efficiency of SrSHT was significantly higher than that of SmSHT (Table 1). Altogether, these data showed that SrSHT had higher activity for acylation of Spd than SmSHT.

## Discussion

Eggplant fruit is highly rich in HCAAs.^21–24^ So far the HCAA biosynthetic pathway has been studied mainly in model plants.^5^ In the current study, we cloned and characterized a putative SHT, SmSHT, from eggplant and SrSHT from the wild relative *S. richardii*. Both of them were able to catalyze the formation of acyl polyamine conjugates. They preferred spermidine to spermine and putrescine for the acyl acceptor (Figures 6b–d), thereby they are primarily spermidine hydroxycinnamoyl transferase. As for acyl donor substrates, both SHTs displayed activity in a preferential order: caffeoyl-CoA>feruloyl-CoA>*ρ*-coumaroyl-CoA, which is similar to a tobacco SHT (NaDH29).^18^ In contrast, *Arabidopsis* AtSHT preferentially utilizes feruloyl-CoA opposed to caffeoyl-CoA and *ρ*-coumaroyl-CoA,^17^ whereas AtSCT and AtSDT exclusively activated *ρ*-coumaroyl-CoA and sinapoyl-CoA, respectively.^4^ Therefore, different plants might develop different acyl donor substrate specificity for SHT enzymes. Mechanism of hydroxycinnamoyl moiety selection of BAHD enzymes has not been thoroughly elucidated. Structural analyses of CcHCT and SbHCT indicate nine residues and one motif potentially involved in acyl donor substrate binding.^19,20^ None of the five substitutions between SmSHT and SrSHT is located in these positions (Supplementary Figure 4). Among the 9 acyl donor binding-related positions, Met-151, which is close to the C-5 hydroxyl of the caffeoyl moieties docked to CcHCT, has been functionally confirmed to be involved in the selection of acyl donor substrate by mutagenesis analysis. If a smaller Val residue replaces the large side chain in this position, extended space may accommodate a methoxyl group on the C-5 hydroxyl of the acyl moieties and increase the binding of ferulic acid.^19^ Met-151 is replaced by Ile in all SHTs, except AtSHT and *Capsicum annuum* SHT (CaSHT), where a Val residue is located (Supplementary Figure 4). Isoleucine has a longer side chain than valine, potentially explaining why both SHTs prefer caffeoyl-CoA, while AtSHT prefers feruloyl-CoA. Another interesting position is Met-406 in SmSHT. Met-406 is conserved in all SHTs, except AtSHT, where a Thr with a short side chain is found (Supplementary Figure 3). Such a small residue could help extend the size of the substrate-binding pocket and thus lead AtSHT to preferentially select feruloyl-CoA. In addition, the Val-31 to Pro-37 loop also forms a part of the hydroxycinnamoyl binding site in CcHCT. If this loop can adopt different conformations to modify the shape of this binding site, a flexible pocket could accommodate various hydroxycinnamoyl moieties including ferulic acid.^19^ Actually, sequences of this loop are highly variable in these proteins with different acyl donor substrate specificities. For example, FAVTHVP is found in both SHTs, GTITHIP in AtSHT, FNEVMYA in AtSCT and YNEVIYK in AtSDT (Supplementary Figure 4). Such variation may contribute to the formation of distinct donor substrate specificities. A site-directed mutagenesis study is needed to elucidate functions of these candidate sites to determine donor substrate specificity.

SrSHT displayed significantly higher enzyme activity than SmSHT as well as other SHTs such as AtSCT (Table 1),^4^ suggesting that this enzyme could be a valuable candidate for manipulating HCAA biosynthesis. SrSHT and SmSHT only contain five residue substitutions (C200S, P286S, R297K, L326S and K402E; Figure 3a). None of these sites overlapped with any of those known catalysis-related positions in SHT isoforms from coffee CcHCT and sorghum SbHCT (Supplementary Figure 4),^19,20^ suggesting that one or more of the five amino acids affect SHT catalytic activity. The most interesting site is K402 because it is adjacent to the conserved motif DFGWG (Figure 3a). A site-directed mutagenesis study has shown that this motif could regulate the catalytic activity by stabilizing the overall two-domain structure of BAHD-like enzymes including maintaining the integrity of the CoA-binding pocket.^20,37,38,39^ Extensive sequence alignment showed that the residue adjacent to the DFGWG motif usually is a lysine (K) or arginine (R) (Supplementary Figure 4). This basic residue was replaced by an acidic and negatively charged glutamic acid (E) in SrSHT, which could potentially modify the conformation of DFGWG motif manifesting in altered catalytic activity of the enzyme. Another interesting change is C200S, located in the crossover loop linking domain I and II of SHT (Figure 3a). Some similar interdomain linkers have been shown to have critical functions during enzyme catalysis. For example, the interdomain linker of an Arabidopsis UDP-glucose transferase could modify the enzyme activity by regulating domain movement, interdomain interaction or activity pocket shape.^40^ In the substitution C200S, cysteine (C) and serine (S) have different chemical properties such hydrophilicity and the formation of disulfide bonds. Actually, a predictive structural change occurred in this exact position. A pair of antiparallel β sheets (Leu206–Leu214) in SmSHT was replaced by short α helices (Ile198–Thr202) in SrSHT (Figure 3c). Such a conformation change in the crossover loop may affect enzyme activity by altering the domain movement, interdomain interaction or activity pocket shape of SHT. As for the other three residue changes (P286S, R297K and L326S), Arg (R) and Lys (K), they are similar residues, while P286S and L326S were located in an unconserved area far from the active site. Therefore, these mutations seem unlikely to have impact on the activity of SHT. Further mutagenesis studies are needed to answer how those mutations will affect the activity of the SHT enzyme.

*SmSHT* was highly expressed in eggplant flowers and fruits. However, the expression level of *SrSHT* in the wild relative was remarkably low. In consistent with the express profile of *SHT* genes, our previous metabolic analysis revealed that *S. melongena* fruit was rich in Spd-HCAAs, whereas the wild relative fruit is rich in Spm-HCAA.^25,41^ We have evidence that there is another highly expressed *SHT* homolog responsible for Spm-HCAA biosynthesis in *S. richardii*. (Peng and Yang, unpublished data). Thus, the difference in the profiles of HCAAs between cultivated and wild eggplant is mainly caused by the transcriptional regulation of the corresponding *SHTs*. It has been suggested that during domestication, the growers preferred fruits with more Spd-HCAAs rather than Spm-HCAAs.

The expression of *SmSHT* was stimulated by heat/drought stress in eggplant leaves and fruits (Figures 4b and c), which is similar to Arabidopsis acyltransferase genes for the biosynthesis of flavonoids or green leaf volatiles that are strongly induced by stress.^16,42^ Some HCAAs have been proved to be able to fight environmental stresses such as scavenging free radicals.^4^ Therefore, it is possible that *in vivo* products of acylation catalyzed by SmSHT may help plant cells tolerate heat stress. Further characterization of SmSHT will shed light on the physiological function of SmSHT.

In conclusion, the current study showed that various CoA esters synthesized by Sm4CL1 could be efficiently converted to polyamine-HCAAs by recombinant SHTs. Therefore, this study replicated the last two steps in HCAA biosynthesis pathway, which not only contributes to our knowledge of HCAA metabolism in plants, but also could be exploited to produce HCAAs in bacteria or yeast expression systems. In addition, the wild-type SrSHT exhibited higher catalytic activity than other SHTs and hence could be used to engineer HCAA metabolism *in planta*, which could improve resistance to biotic/abiotic stresses and enhance the nutritional quality of agricultural produce for human health benefits. Furthermore, our sequence and enzymological analysis provided two promising candidate sites (Cys-200 and Lys-402) for studying the catalytic mechanism of SHTs.

## Figures and Tables

**Table 1:** Kinetic parameters of SmSHT and SrSHT with fixed concentrations of spermine and varying acyl donor substrates

*Acyl donor*	*Enzyme*	*K*_*cat*_ *(min*^*−1*^)	*K*_*m*_ *(μm)*	*K*_*cat*_*/K*_*m*_ *(min*^*−1*^ *μm*^*−1*^)
Caffeoyl-CoA	SmSHT	441.8±11.1^b^	58.3±1.7^a^	7.6±0.2^b^
	SrSHT	606.8±29.0^a^	67.1±7.6^a^	9.0±0.4^a^
Feruloyl-CoA	SmSHT	232.1±12.3^d^	27.3±5.6^c^	8.5±0.5^a^
	SrSHT	413.0±60.0^b^	51.3±10.6^a,b^	8.0±1.2^a,b^
*ρ*-Coumaroyl-CoA	SmSHT	181.2±7.3^e^	29.6±5.8^b,c^	6.1±0.2^d^
	SrSHT	259.7±14.6^c^	38.4±3.3^b^	6.8±0.4^c^

Different letters (a, b, c) in all the parameters indicate significant differences among mean values (*P*<0.05; *t*-test).
