# Supplementary figures and images for: Characterization of spermidine hydroxycinnamoyl transferases from eggplant (Solanum melongena L.) and its wild relative Solanum richardii Dunal
Source: Hortic Res. 2016 Dec 7;3:16062–. doi: 10.1038/hortres.2016.62 (PMC5142293; doi:10.1038/hortres.2016.62)

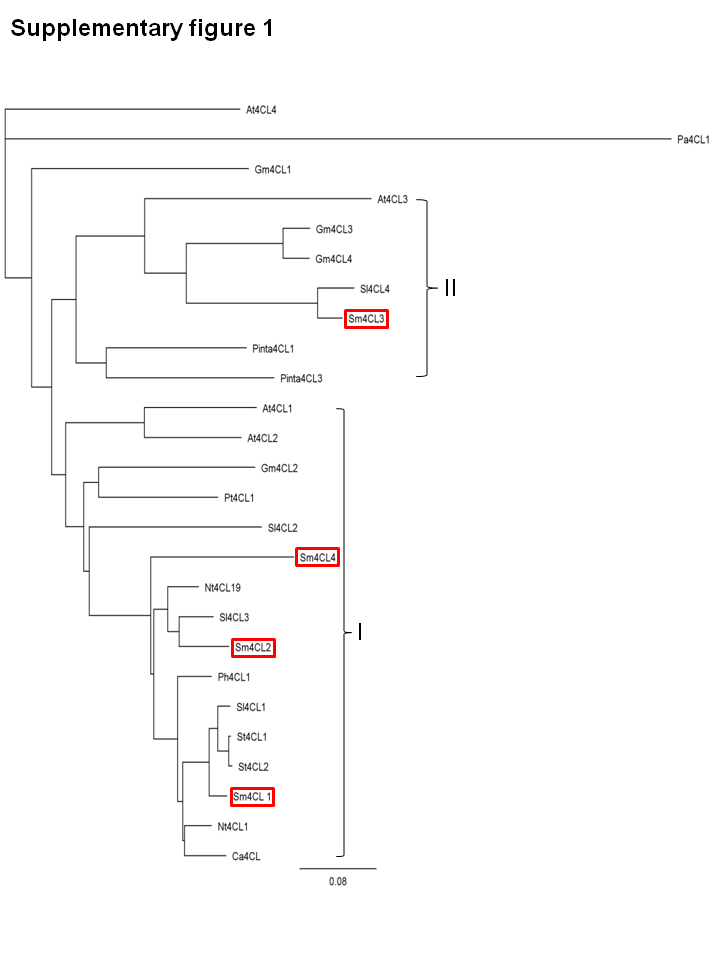

Supplement: Supplementary Figure 1 [file hortres201662-s1.tiff]

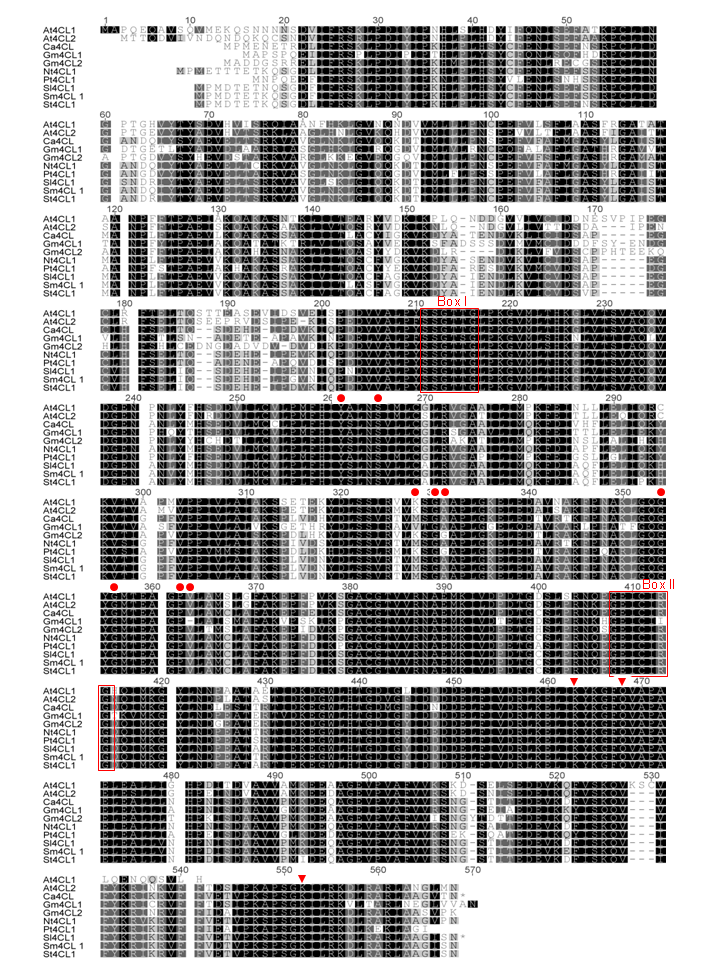

Supplement: Supplementary Figure 2 [file hortres201662-s2.tiff]

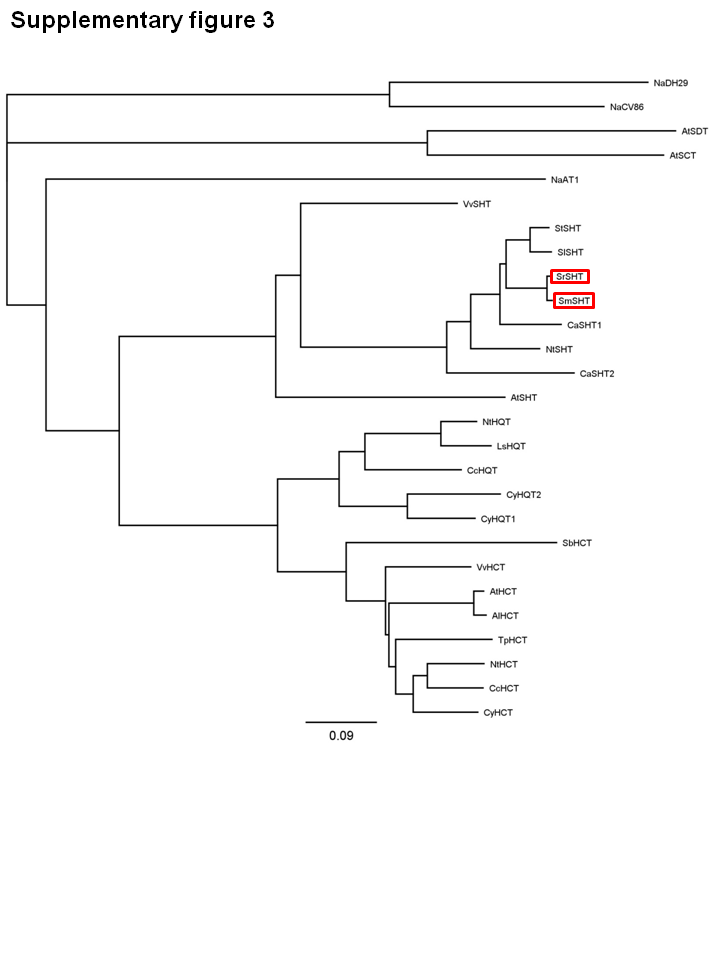

Supplement: Supplementary Figure 3 [file hortres201662-s3.tiff]

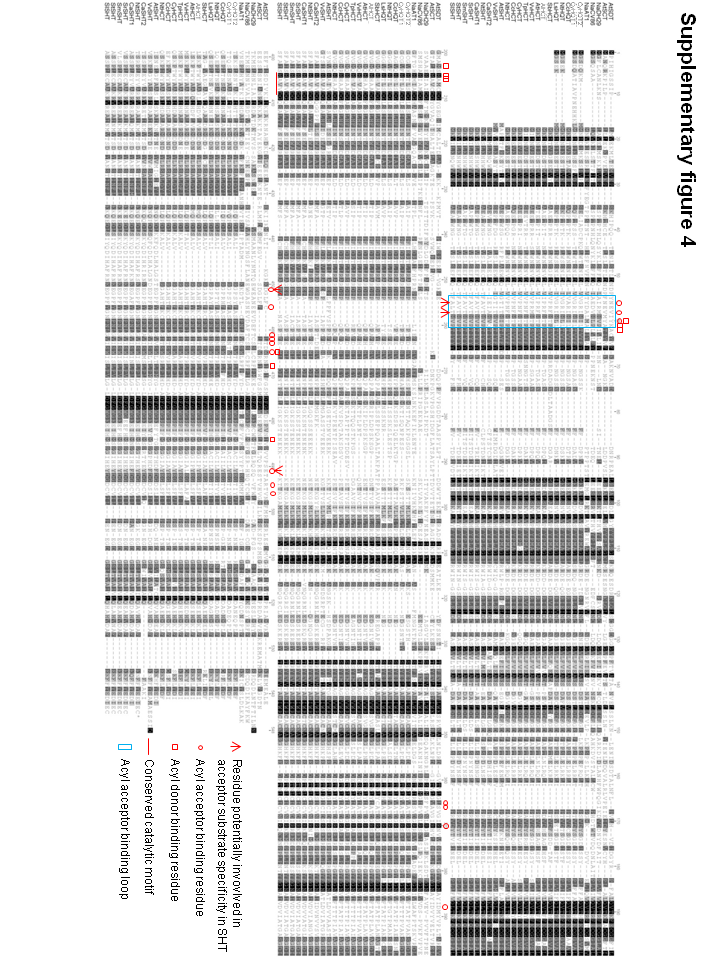

Supplement: Supplementary Figure 4 [file hortres201662-s4.tiff]

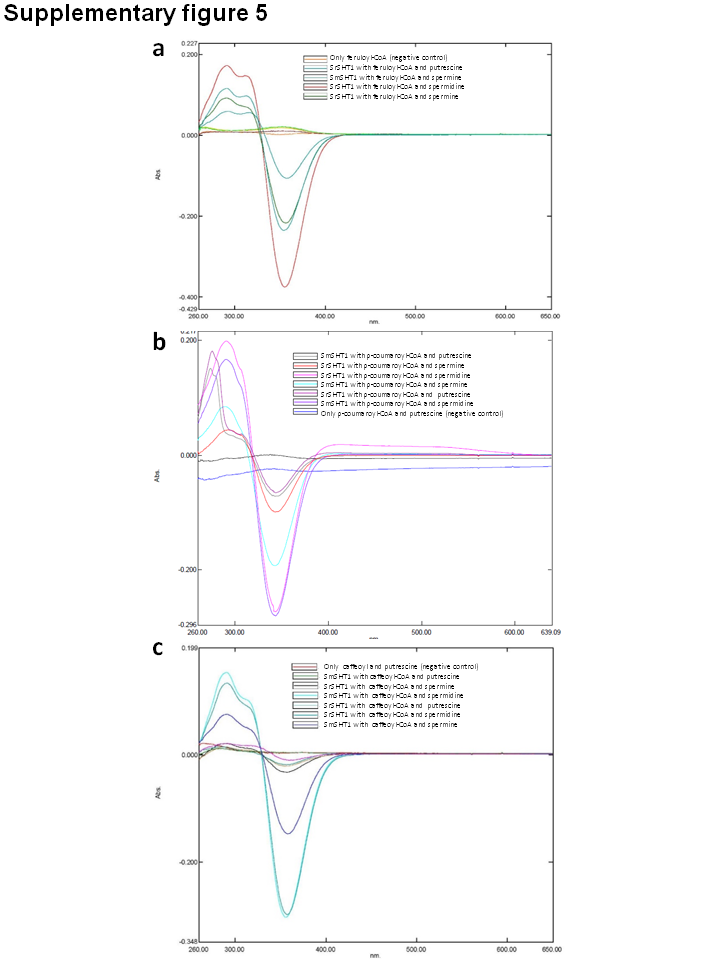

Supplement: Supplementary Figure 5 [file hortres201662-s5.tiff]

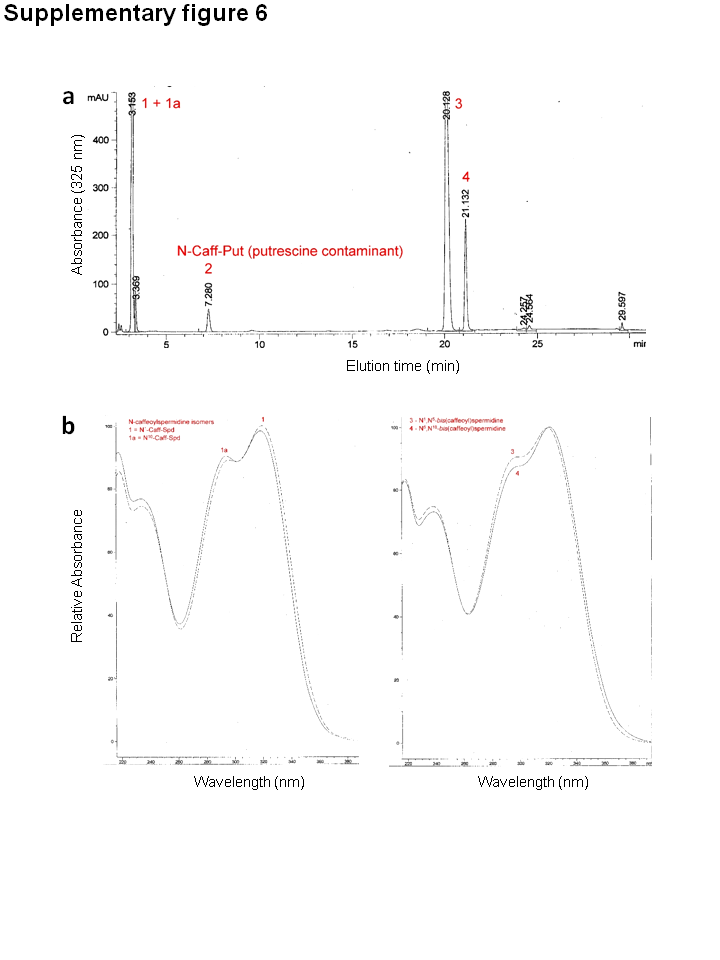

Supplement: Supplementary Figure 6 [file hortres201662-s6.tiff]

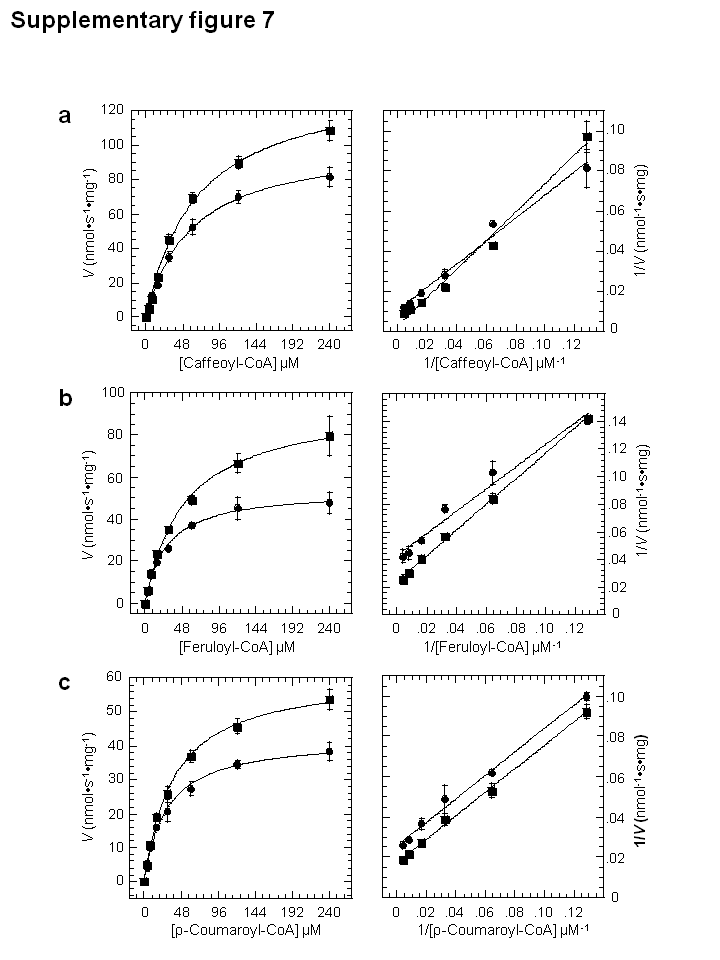

Supplement: Supplementary Figure 7 [file hortres201662-s7.tiff]
