# Supplementary material for: Characterization of spermidine hydroxycinnamoyl transferases from eggplant (Solanum melongena L.) and its wild relative Solanum richardii Dunal
Source: Hortic Res. 2016 Dec 7;3:16062–. doi: 10.1038/hortres.2016.62 (PMC5142293; doi:10.1038/hortres.2016.62)
Supplement: Supplementary Figure Legends [file hortres201662-s8.doc]

**Supplementary figure legends**

**Supplementary figure 1**. Phylogenetic analysis of Sm4CLs and other 4CL proteins. The bar indicates the relative change between each branch. Eggplant 4CLs are marked by open red boxes. *Arabidopsis thaliana* At4CL1 (AT1G51680), At4CL2 (AT3G21240), At4CL3 (AT1G65060), At4CL4 (AT3G21230), *Capsicum annuum* Ca4CL (CA03g30500), *Glycine max* Gm4CL1 (AF279267), Gm4CL2 (AF002259), Gm4CL3 (AF002258), Gm4CL4 (X69955), *Nicotiana tabacum* Nt4CL1 (AAB18637), Nt4CL19 (AAB18638), *Pelargonium appendiculatum* Pa4CL1 (KJ944317), *Pinus taeda* Pinta4CL1 (P41636), Pinta4CL3 (AGX45528), *Petunia x hybrid* Ph4CL1 (JN120849), *Populus tomentosa* Pt4CL1 (AAL02145), *Solanum lycopersicum* Sl4CL1 (AK328438), Sl4CL2 (XP_004242790), Sl4CL3 (XP_004241647), Sl4CL4 (XP_004235294), *Solanum melongena* Sm4CL1 (KP892652), Sm4CL2 (Sme2.5_00008.1_g00010.1), Sm4CL3 (Sme2.5_00843.1_g00005.1), Sm4CL4 (Sme2.5_02524.1_g00005.1), *Solanum tuberosum* St4CL1 (P31684), and St4CL2 (P31685).

**Supplementary figure 2**. Multiple amino acid sequence alignment of Sm4CL1 with other plant 4CLs. Boxed amino acids represent conserved motifs. Residues involved in hydroxycinnamate binding are indicated by red circles on top, while those involved in enzymatic function are marked by red triangles. *Arabidopsis thaliana* At4CL1 (GenBank accession AT1G51680), At4CL2 (AT3G21240), *Capsicum annuum* Ca4CL (CA03g30500), *Glycine max* Gm4CL1 (AF279267), Gm4CL2 (AF002259), *Nicotiana tabacum* Nt4CL1 (AAB18637), *Populus tomentosa* Pt4CL (AAL02145), *Solanum lycopersicum* Sl4CL1 (AK328438), *Solanum tuberosum* St4CL1 (P31684), and *Solanum melongena* Sm4CL1 (KP892652).

**Supplementary figure 3.** Phylogenetic analysis of eggplant SHTs with other BAHD-containing proteins. The bar indicates the relative amount of change along each branch. Eggplant SHTs are marked by open red boxes. *Arabidopsis lyrata* AlHCT (EFH70827), *Arabidopsis thaliana* AtHCT (AT5G48930), AtSCT (AT2G25150), AtSDT (AT2G23510), AtSHT (AT2G19070), *Capsicum annuum* CaSHT1 (Capana00g001430), CaSHT2 (Capana05g000927),*Coffea canephora* CcHCT (EF137954), CcHQT (EF153931), *Cynara cardunculus* CyHCT (DQ104740), CyHQT1 (EU697935), CyHQT2 (EU839580), *Lycopersicon esculentum* LsHQT (AJ582652), *Nicotiana attenuata* NaDH29 (JN390824), NaCV86 (JN390825), NaAT1 (JN390826),*Nicotiana tabacum* NtSHT (unannotated), NtHQT (AJ582651), NtHCT (CAD47830), *Sorghum bicolor* SbHCT (XP_002452435), *Solanum lycopersicum* SlSHT (Solyc07g015960.1.1),*Solanum melongena* SmSHT (KP165410), *Solanum richardii* SrSHT (KP165411), *Solanum tuberosum* StSHT (PGSC0003DMP400059459), *Trifolium pratense* TpHCT (EU861218),*Vitis vinifera* VvHCT (XP_002268988), and VvSHT (XP_002269790).

**Supplementary figure 4**. Multiple amino acid sequence alignment of eggplant SHTs with other BAHD-domain containing polypeptides. *Arabidopsis lyrata* AlHCT (EFH70827), *Arabidopsis thaliana* AtHCT (AT5G48930), AtSCT (AT2G25150), AtSDT (AT2G23510), AtSHT (AT2G19070), *Capsicum annuum* CaSHT1 (Capana00g001430), CaSHT2 (Capana05g000927), *Coffea canephora* CcHCT (EF137954), CcHQT (EF153931), *Cynara cardunculus* CyHCT (DQ104740), CyHQT1 (EU697935), CyHQT2 (EU839580), *Lycopersicon esculentum* LsHQT (AJ582652), *Nicotiana attenuata* NaDH29 (JN390824), NaCV86 (JN390825), NaAT1 (JN390826),*Nicotiana tabacum* NtSHT (unannotated), NtHQT (AJ582651), NtHCT (CAD47830), *Sorghum bicolor* SbHCT (XP_002452435), *Solanum lycopersicum* SlSHT (Solyc07g015960.1.1),*Solanum melongena* SmSHT (KP165410), *Solanum richardii* SrSHT (KP165411), *Solanum tuberosum* StSHT (PGSC0003DMP400059459), *Trifolium pratense* TpHCT (EU861218), *Vitis vinifera* VvHCT (XP_002268988), and VvSHT (XP_002269790).

**Supplementary figure 5.** Spectrophotometric analysis of SmSHT and SrSHT acylation reactions. (a) Spectrophotometric chart of acylations with feruloyl-CoA and various polyamines (spermidine, spermine, and putrescine) catalyzed by SmSHT and SrSHT. (b) Spectrophotometric chart of acylations with ρ-coumaroyl-CoA and various polyamines (spermidine, spermine, and putrescine) catalyzed by SmSHT and SrSHT. (c), Spectrophotometric chart of acylations with caffeoyl-CoA and various polyamines (spermidine, spermine, and putrescine) catalyzed by SmSHT and SrSHT.

**Supplementary figure 6.**  (a) C18-HPLC-DAD chromatogram of reaction products catalyzed by SmSHT with spermidine and caffeoyl-CoA (mAU, milli-absorption units). (b) UV absorbance spectra of mono- (peaks 1a-c), bis- (peaks 2a and 2b), and tris- (peak 3) N-feruloylspermidine conjugates.

**Supplementary figure 7.** SHT kinetics with varying hydroxycinnamoyl-CoA concentrations under fixed Spd. Data were fitted to the Michaelis–Menten equation shown on the left. The Lineweaver-Burk plot is displayed on the right. Each point represents the mean velocity plus SD from triplicate reactions ( SmSHT; SrSHT).
